# Legislation

**Published:** 1898-05

**Authors:** 


### LEGISLATION.

### New York.

Assembly bill 979, known as the "bob-veal" bill, is for the purpose of preventing the sale for food of calves or carcasses of calves under four weeks old. This is believed to be in the interest of the consuming public, and will result in financial good to New York farmers. It is opposed by no one except dealers in "bob-veal" in the large cities and men who have been inspired by them. The benefit to the farmers will come from the fact that the calves which are now sold for veal at ages varying from a half-hour upward, would be kept at the farm and raised to the proper age and fattened, and would consume a sufficient quantity of milk, so as to relieve what is generally known at a certain time of the year to dealers as glut in the milk-market. These calves of the proper age would probably take the milk of 75,000 cows off the market, thereby producing meat from these calves that would be healthful and wholesome, and in which the consuming public would have confidence, so that veal would be more freely eaten in restaurants and hotels, thereby making a market for more farm produce from this State.

Assembly bill 1511 was approved by the department of agriculture at the request of milk-producers, or those representing them, in that portion of the State sending milk to New York and Brooklyn. A sentiment has been created during the past few years to the effect that there were men who might be prosecuted for violation of the law in the milk that they were selling, when it was just as it came from the cow. This law provides, first, that before taking a sample the agent of this department should call upon the vendor to stir thoroughly and mix the milk, and if he refuses then the agent is to do so. If the sample taken when analyzed is found below this standard, then this department is required to send a person within ten days' time to take a fair sample of the mixed milk from the herd from which the milk was drawn, taking it in the same way that the original sample was taken, and if upon analysis the second sample is not shown to contain a higher percentage of solids and a higher percentage of butter-fats, then no action lies against the defendant. We believe this in one sense is no hardship, although it entails a little more work, for we believe that the mixed milk from herds of cows will not fall below the standard, but it is in response to public sentiment, and will probably facilitate our prosecutions in that section of the State.

The Empire State will test a new road-law destined to improve the public highways, the State paying 25 per cent. as much as is levied for road purposes by the districts taking advantage of the law.

Under new legislative enactments the Governor will appoint two supervisors and collectors of the tax imposed on racing, trotting, and running tracks. Said supervisors to examine the books and records of the tracks and to receive salaries of $2500 per year. Charles W. Anderson has been appointed supervisor of the running tracks.

A ringing bill has been passed and signed by the Governor, thus making it a misdemeanor to enter for any race an animal under any other than its proper name.

---

DEHORNING AND CASTRATION.—J. M. B., Leslie, N. Y.: "I am a farmer and have dehorned cattle and altered colts for six years. Now the veterinarians say I have no right to do it, only as I own the stock. I have not been able to find out just what the law is." Answer: As to the law you can see a copy of it by going to the county clerk's office. Under its provisions you have no right to practise either dehorning or castrating. While I do not like to advise anyone to do an unlawful act, I will say that a profession that needs to get such a law passed to protect it is indulging in mighty low business. If you are a successful man in the line of castration and dehorning animals, and a farmer wants you to do it for him, you have the right to do it; but you cannot charge for doing it, neither can you advertise yourself as a practitioner of the *business* unless licensed to do so. But if your neighbor wishes it done you have the right to do it, and then if he does not make you a present of a sum of money equivalent to its worth to him he is a meaner man than even the veterinarian who would try to prevent your doing it. You cannot collect any pay under the law, but who wants to collect anything? Honorable men expect to pay for services rendered, and will. As I travel over the State in farmers' institute work I find no reputable veterinarians who feel at all like trying to enforce the law. It is those who have but little knowledge of the science who want law to protect them. They, of course, are howling to have laws passed to aid them, yet claim that it is the farmer who is protected from impostors by this law. But in most cases whenever they look in the mirror they will see the image of one who would fleece the farmer for more than the fellows who castrate animals or dehorn them. I think I can safely say that the law among reputable veterinarians of the State is a dead letter, and public opinion is certainly against it, and no one who castrates and dehorns in a proper and humane manner need have much fear of prosecution under the law.—Veterinarian Smead, in the *National Stockman and Farmer*, February 24, 1898.

It would be well to know who this large number of veterinarians are, and if their education was received from the same sources as the veterinarian of the *Stockman and Farmer*. Such evidences of appreciation of the value of higher veterinary education as implied above are in keeping with the subjoined letter from an existing practitioner in Pennsylvania. Further comment is unnecessary.

The following letter from one of Pennsylvania's "existing practitioners" was received in reply to a request to fill out a blank that was sent to all of the registered veterinary surgeons in the State:

S———, February 21, 1898.

DEAR SIR: I git your letter in hand Last week for to fill out this Plank Now I wish you would lit me know wich veterinary surgeons tells you About me I wish you would sent me the law that would Compel me to fill out that Plank Now I will tell you I did not graduate I handle medicine since sixty nine and Some before that time but not so regular Just since seventy nine I bin regular in this bisness and and Just so much to Do as annyone from the graduated and I used the Humphreys Specifice since 1882 with the old school medicine I treat over thirty four hundred horses with lame and all kind sickness and lost only twenty three horses that is all what I have to say Just now I Registered in 1890 P———.

Yours Truly

